# Spur Cells Causing Severe and Transfusion-Refractory Anemia in Patients With Acute-on-Chronic Liver Failure

**DOI:** 10.7759/cureus.10568

**Published:** 2020-09-21

**Authors:** Chandan kumar Kedarisetty, Ramesh Kumar

**Affiliations:** 1 Hepatology, Sri Ramachandra Institute of Higher Education and Research, Chennai, IND; 2 Gastroenterology, All India Institute of Medical Sciences, Patna, IND

**Keywords:** anemia, alcohol, cirrhosis, aclf, acanthocytes

## Abstract

Acute-on-chronic liver failure (ACLF) is characterized by acute decompensation of chronic liver disease associated with organ failures. Anemia of diverse etiology is common in patients with ACLF. Spur cell anemia (SCA) is a form of acquired hemolytic anemia that occurs rarely in such patients due to dysregulated lipids metabolism. Spur cells are large erythrocytes with spike-like projections, which predispose them for sequestration and destruction in splenic canaliculi. There is a paucity of data on SCA in patients with ACLF. Here we report a series of five ACLF patients who had severe (hemoglobin level < 8 g/dL) and transfusion-refractory SCA with aggressive clinical course and high mortality rate.

## Introduction

An adequate oxygen supply is critical to maintain organ function especially in severely ill patients. A generalized decrease in oxygen-carrying capacity due to anemia can lead to an increase in morbidity and mortality in such patients. Acute-on-chronic liver failure (ACLF) is characterized by acute decompensation of chronic liver disease associated with organ failures and high short-term mortality. Anemia is a common finding in patients with ACLF [1]. Moreover, anemia has been found to be an independent predictor for the development of ACLF in patients with chronic liver disease [2]. The etiology for anemia in cirrhosis is usually multifactorial, with the commonest causes including gastrointestinal hemorrhage, hypersplenism, and nutritional deficiencies [1]. Spur cell anemia (SCA) has shown to be associated with advanced liver disease; however, it is generally under-reported [3-5]. Spur cells or acanthocytes are large red blood cells (RBCs) with spike-like projections resulting in deformed shape and flexibility, which predispose them to sequestration and destruction in splenic canaliculi. Patients with SCA usually present with acquired hemolytic anemia [6]. Spur cells occur as a result of increased cholesterol-to-phospholipid ratio in the RBC membrane [7]. There is a paucity of data on the clinical implications of SCA in the natural history of ACLF. We report here a series of five ACLF patients who had severe and refractory SCA and discuss the relevant literature.

## Case presentation

**Figure 1:**
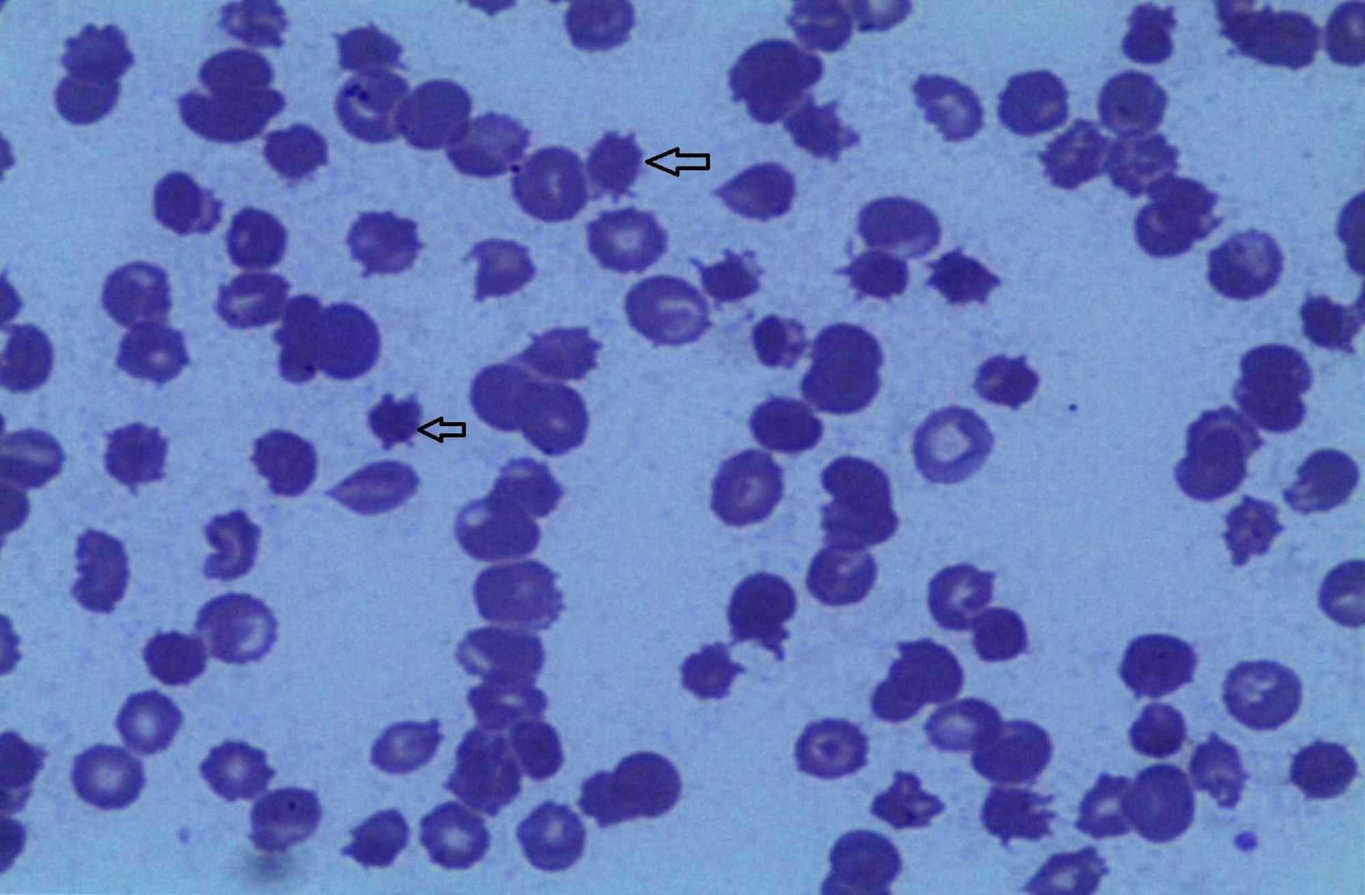
Peripheral blood smear of a patient, showing the presence of multiple spur cells.

The characteristics of all five ACLF patients with severe anemia are given in Table 1. All patients were males, with age varying between 31 and 49 years. The etiology of underlying chronic liver disease was alcohol in all, whereas the acute precipitants were acute hepatitis E in two patients and heavy alcohol consumption in the rest three. All patients had advanced liver disease with marked coagulopathy. The Child-Pugh scores of patients varied from 12 to 14, and the Model for End-Stage Liver Disease (MELD) score varied from 30 to 35. The hemoglobin levels in them ranged from 6.4 to 7.3 g/dL, and there was an evidence of hemolysis in the form of increased blood levels of reticulocyte count, lactate dehydrogenase, and indirect bilirubin. A peripheral blood smear of all patients revealed poikilocytosis with the presence of numerous (>5%) spur cells (Figure 1). None of the patients had current or recent gastrointestinal bleeding, pancytopenia indicating hypersplenism, Coombs positivity, and iron or vitamin B12 deficiency. The anemia of all patients was persistent and refractory to multiple blood transfusions due to non-sustained improvement in hemoglobin levels. Evaluation of serum lipid profiles revealed markedly reduced levels of total cholesterol, low-density lipoprotein (LDL) cholesterol, high-density lipoprotein (HDL) cholesterol, and triglyceride in all five patients. In-hospital course of four patients was very aggressive as they developed hepatic encephalopathy and acute kidney injury; three patients had variceal bleeding, and one patient developed diffuse alveolar hemorrhage. Liver transplantation could not be carried out in any of them due to rapid clinical deterioration and logistic reasons. Three of them died in the hospital, and one was taken to another hospital in a serious condition against medical advice. Only one patient (case 1) showed improvement in ACLF and subsequently got discharged from the hospital. Though mild non-SCA persisted in him with hemoglobin levels over 9 g/dL, he became transfusion-independent and was doing fine up until three months of follow-up.

**Table 1:** Characteristics of ACLF patients with SCA Abbreviations: ACLF, acute-on-chronic liver disease; ALT, alanine transaminase; AST, aspartate transaminase; HDL, high-density lipoprotein; Hep enceph, hepatic encephalopathy; INR, international normalized ratio; LDH, lactate dehydrogenase; LDL, low-density lipoprotein; MCH, mean corpuscular hemoglobin; MCHC, mean corpuscular hemoglobin concentration; MCV, mean corpuscular volume; MELD, Model for End-Stage Liver Disease; SCA, spur cell anemia

Parameters	Case 1	Case 2	Case 3	Case 4	Case 5
Age (years)	49	31	35	47	31
Gender	Male	Male	Male	Male	Male
Etiology of ACLF (acute/chronic)	Alcohol/alcohol	Alcohol/alcohol	Hepatitis E/alcohol	Hepatitis E/alcohol	alcohol/alcohol
Child-Pugh Score	13	14	13	12	12
Hemoglobin (g/dL)	6.6	6.4	6.7	6.6	7.3
MCV (fL/cell)	105	105	96	100	99
MCH (pg/cell)	34	36	35	37	36
MCHC (g/dL)	34	35	36	37	36
Reticulocyte count (%)	1.9	2.27	6.3	3.1	2.3
Total leukocyte count/cmm	14000	8800	9100	6200	30300
Platelets count/cmm	129000	50000	40000	70000	208000
Serum LDH (U/L)	330	765	880	610	702
Serum ferritin (ng/mL)	601	363	2130	224	446
Serum vitamin B12 (ng/mL)	1500	1277	714	960	1063
Serum total bilirubin (mg/dL)	29.5	19.7	35.3	34.8	31.5
Indirect bilirubin (mg/dL)	14.9	11.6	19.3	18.5	17
Serum AST (IU/L)	62	60	99	161	133
Serum ALT (IU/L)	36	41	93	92	25
Serum albumin (mg/dL)	3.3	3.6	2.2	2.8	2.7
INR	2.5	2.9	3.5	3.0	3.9
Serum Creatinine (mg/dL)	1.18	1.1	0.7	0.6	0.7
Serum total cholesterol (mg/dL)	37	34	38	63	42
Serum LDL cholesterol (mg/dL)	10	8.6	5.7	19	13
Serum HDL cholesterol (mg/dL)	05	10.7	7.1	12	11
Serum Triglyceride (mg/dL)	51	30	34	32	35
MELD score	31	30	34	32	35
In-hospital complications	Hep enceph, AKI	Hep enceph, AKI, diffuse alveolar hemorrhage	Hep enceph, AKI, variceal bleeding	Hep enceph, variceal bleeding	Hep enceph, variceal bleeding AKI
Hospital outcome	Discharged	Died	Died	Discharged (against medical advice)	Died

## Discussion

SCA in patients with advanced liver disease is frequently overlooked. Though there is a paucity of data on SCA, it has been described in patients with alcoholic cirrhosis [8,9], cholestatic liver disease [10,11], and post-liver transplant allograft failure [12]. To the best of our knowledge, ours is the first case series of severe SCA in patients with ACLF. Although spur cells may be present in those patients before the onset of acute decompensation, severe anemia was noted only after the development of ACLF.

The mechanistic pathogenesis of SCA in patients with advanced liver disease involves an imbalance in lipid metabolism that affects the fluidity and lipid composition of RBC membranes. In spur cells, the ratio of cholesterol to phospholipid is increased in the membrane [7]. With progressive deterioration of liver functions, there is also a progressive decline in the serum levels of total cholesterol, LDL cholesterol, and HDL cholesterol [13]. Moreover, the study has found that in cirrhosis patients with SCA, the levels of apolipoprotein A-II are significantly reduced than in cirrhosis patients without SCA [14]. This causes a significant alteration in the structure and metabolism of HDL fraction. The dysregulated lipid metabolism in patients with advanced liver disease appears to affect in vivo survival of transfused RBCs as well, which often results in transfusion refractory anemia. In our study, all patients with SCA had markedly reduced levels of serum lipids. One study has found a partial improvement in serum lipids after plasmapheresis [15]. Though all patients with SCA had higher reticulocyte count, it was not proportionately high, suggesting some degree of ineffective erythropoiesis due to advanced liver disease and alcoholism [16].

The presence of SCA is significantly associated with the severity of liver disease, increased in-hospital bleeding complications, hepatic encephalopathy, organ failures, and poor outcome. In the study, the three-month survival was 60% in those with spur cells compared to 92.3% in those without spur cells [3]. Sousa et al. reported an overall survival of one month, highlighting the significance of the presence of spur cells on prognosis in advanced liver disease [8].

Treatment options for SCA are limited and disappointing. There is one report on the use of high-dose steroids for reducing hemolysis, but one needs to be cautious in ACLF patients due to the inherent risk of secondary infections [17]. Plasmapheresis has been shown to reduce hemolysis to a certain extent and partially improve serum lipids; however, it has no effects on the magnitude of spur cells [14]. The only recommended treatment is liver transplantation, with documented reversibility of SCA [18].

## Conclusions

In conclusion, spur cells can cause severe and refractory anemia in patients with ACLF. The presence of SCA is associated with increased liver severity scores, increased risk of bleeding complications, and higher overall short-term mortality rates. Early recognition of this condition and timely liver transplantation can improve the outcome of such patients.
